# Suboptimal care and maternal mortality among foreign-born women in Sweden: maternal death audit with application of the ‘migration three delays’ model

**DOI:** 10.1186/1471-2393-14-141

**Published:** 2014-04-12

**Authors:** Annika Esscher, Pauline Binder-Finnema, Birgit Bødker, Ulf Högberg, Ajlana Mulic-Lutvica, Birgitta Essén

**Affiliations:** 1 Department of Women’s and Children’s Health, International Maternal and Child Health (IMCH), Uppsala University, SE-751 85 Uppsala, Sweden; 2 Department of Obstetrics and Gynaecology, Hillerød Hospital, Hillerød, Denmark

**Keywords:** Maternal migration effect, Foreign-born, Structured implicit review, Maternal death audit, Low-income country

## Abstract

**Background:**

Several European countries report differences in risk of maternal mortality between immigrants from low- and middle-income countries and host country women. The present study identified suboptimal factors related to care-seeking, accessibility, and quality of care for maternal deaths that occurred in Sweden from 1988–2010.

**Methods:**

A subset of maternal death records (n = 75) among foreign-born women from low- and middle-income countries and Swedish-born women were audited using structured implicit review. One case of foreign-born maternal death was matched with two native born Swedish cases of maternal death. An assessment protocol was developed that applied both the ‘migration three delays’ framework and a modified version of the Confidential Enquiry from the United Kingdom. The main outcomes were major and minor suboptimal factors associated with maternal death in this high-income, low-maternal mortality context.

**Results:**

Major and minor suboptimal factors were associated with a majority of maternal deaths and significantly more often to foreign-born women (p = 0.01). The main delays to care-seeking were non-compliance among foreign-born women and communication barriers, such as incongruent language and suboptimal interpreter system or usage. Inadequate care occurred more often among the foreign-born (p = 0.04), whereas delays in consultation/referral and miscommunication between health care providers where equally common between the two groups.

**Conclusions:**

Suboptimal care factors, major and minor, were present in more than 2/3 of maternal deaths in this high-income setting. Those related to migration were associated to miscommunication, lack of professional interpreters, and limited knowledge about rare diseases and pregnancy complications. Increased insight into a migration perspective is advocated for maternity clinicians who provide care to foreign-born women.

## Background

Differences in maternal mortality between immigrants and host country women are observed in several European countries, often with elevated risk for women coming from low-income countries (LIC) or middle-income countries (MIC) [1-4]. In Sweden during 1988–2007, the maternal mortality ratio for all women was 6 per 100 000 live births, for Swedish-born women 5.9, for women born in LIC 21.1, MIC 4.7, and other high-income countries (HIC) 5.5 [5,6]. Over the past decade, Confidential Enquiries into maternal deaths in the United Kingdom (UK) have consistently shown that maternal deaths among ‘Black’ African mothers, including women from LIC settings in sub-Saharan Africa, are significantly more prevalent and have more frequently resulted from direct causes relative to ‘White’ British-born women [7,8].

Why women from lower income and low-resource settings are at higher risk of dying than host European women cannot be easily explained by well-known obstetric and socioeconomic risk factors [1,8,9]. However, maternity care provided to foreign-born women is frequently reported as substandard, especially when it comes to availability of medical translation services and culturally competent care providers, and for underestimation of an existing condition due to communication barriers [1,2,8,10,11]. Averting maternal mortality may thus be dependent upon provision of optimal emergency care [12] that is provided in a timely way [13].

The necessity of recording the number and causes of death is not in question [14]. Accurate maternal mortality surveillance is considered an important tool for providing appropriate and effective patient care [8]. However, in HIC maternal mortality is rare, and it can take years to approach a clear understanding about risks and how to improve health care conditions in a single, particular setting [7,15]. Scrutinising the road to death may therefore clarify why the incidence is higher for different groups of women [4,16,17] and why women representing high risk groups face barriers to care-seeking or regular utilisation of available maternity care services [8,18,19]. Assessing both the quality of maternity care that a woman received and her own pregnancy care strategies may be essential [20].

In this study, we aimed to identify suboptimal factors of maternity care related to maternal death as it occurred in Sweden over a period of increased migration of childbearing women from LIC and MIC [21]. Our specific objectives were to quantify the medical causes of maternal death, and to explore these in relation to clinical care and sociocultural influences. This approach attempts to unravel the paradox of maternal death in a high-income, social medical system that offers open access to all pregnant women who live in the country.

## Methods

### Data collection

Maternal death data were collected from Swedish official and national registries (n = 123) for 1988 to 2007 [5], and cases from the Swedish Society of Obstetrics and Gynaecology (SFOG) Maternal Mortality Group for 2008 to 2010 (n = 17). We have defined maternal mortality according to the ICD-9 and ICD-10 definition as “the death of a woman while pregnant or within 42 days (that is, between Day 0 and 41) of termination of pregnancy – irrespective of the duration and site of the pregnancy – from any cause related to or aggravated by the pregnancy or its management, but not from accidental or incidental causes” [22]. Women’s country of birth was classified according to the World Bank [23]. We then selected all maternal deaths from LIC and MIC during 1988–2010 (n = 26). One of these cases was excluded because the record was not retrievable from the hospital archive. Immigrants from high-income countries were excluded because they either came from other Nordic countries, which have similar languages and health care systems to the Swedish, or from Anglophone countries, which implied that language barriers would be difficult to detect because most Swedish care providers are proficient in English. For each of the remaining 25 foreign-born maternal deaths, two Swedish-born cases were matched by gestational age (with a cut-off point at 24 weeks) and day of death. That is, if a foreign-born woman died before reaching gestational age 24 weeks, she was matched to two Swedish-born cases having died before week 24, as in the case closest before and after the death of the index person. Since there was a cluster of foreign-born women who died towards the end of the study period, we could not always find a Swedish-born woman dying after the index case. To compensate, we chose two cases that died earlier. The medical records comprised all available information about a deceased woman’s treatment and came from the hospitals where she either died or received treatment before her death. The latter included information from the antenatal and delivery records from the index pregnancy. Data used for analysis comprised 75 maternal death records.

Two obstetricians independently reviewed each case file using a version of the “Maternal Deaths Enquiry” developed by CMACE, which was modified for this study to reflect the Swedish context. Maternal death in Sweden has not been systematically reviewed by the Swedish Maternal Mortality Group prior to 2007, when it was formed. The present dataset therefore reflect only those data based on medical records and not, for example, on interviews conducted with hospital staff or those medical professionals associated with a particular case. Following the initial review, the additional members of the audit group were provided a copy of the case assessments and a narrative summary of each case, which was anonymised by the first author. Each narrative summary contained a description of time-related events relevant to pregnancy, delivery and the postpartum period, and included a woman’s medical history, the available information about her social conditions and cultural background, her language skills, as well as the surveillance and interventions she received, and the death outcome. These narratives were derived precisely from the records and any missing pertinent information was duly noted.

### Audit protocol: medical and sociocultural factors

**Figure 1:**
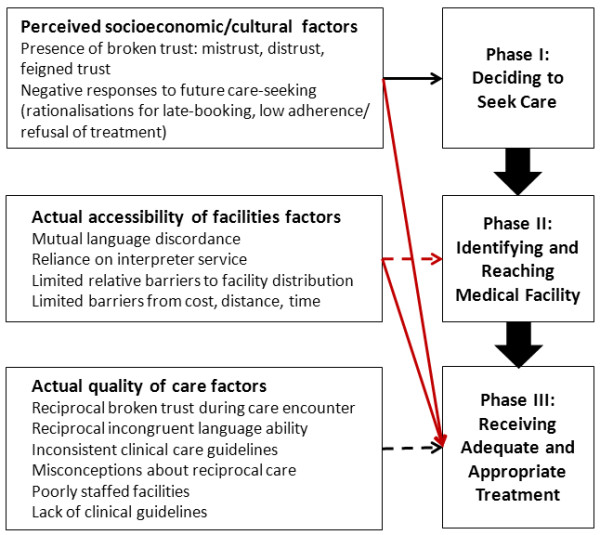
**Factors influencing care-seeking and utilisation of facility-based care and obstetric outcome in high-income western settings **[26]**.**

The structured implicit review [24], which identified major and minor factors of suboptimal care, began with the development of a coded audit protocol. This protocol applied a modified version of the ‘three delays’ model [25], referred to as the ‘migration three delays’ model of Binder et al. [26]. The model assumes a ‘maternal migration effect’ where a woman’s pre-migration experiences in LIC may negatively influence her care-seeking and utilisation following migration to a HIC host country [27]. Phase 1 involves all the social factors underlying a woman’s decision to seek emergency or non-emergency care. Phase 2 facilitates a woman’s ability to identify and reach a medical facility. Phase 3 comprises all the factors that allow a woman to receive adequate and appropriate treatment for a suspected obstetric complication. The context in Sweden is that facility-based care and childbirth were the norms of childbearing during the years represented by the dataset. The model thus provides a theoretical conceptual framework to understand how immigrant women’s sociocultural aspects might have created barriers to their receipt of optimal care in this setting. Moreover, the model can also help to facilitate insight into how HIC maternity care professionals provided care to immigrant women during these years. The flexibility of the model is that factors identified by it can be made context-specific to any high-income setting. Figure 1 illustrates the sociocultural factors that can influence maternity care-seeking and utilisation in European high-income settings [27].

### The procedure

The severity of suboptimal factors was assessed as follows. A factor was labelled as minor if it was a relevant contributory factor and an alternative management strategy might have made a difference to the outcome, but the mother’s survival was unlikely in any case. A factor was labelled as major if it contributed significantly to the death of the mother, and if the death could have most likely been avoided by different management of the case [8]. According to the migration ‘three delays’ model, one woman could have experienced several suboptimal events within the same level of delay. For a case to be assessed as major suboptimal care, at least one of these had to be a major contributing factor. Likewise, suboptimal care would be assessed as minor if there were no major but at least one minor suboptimal factor. If the summary transcripts failed to allow unanimous consensus of suboptimal factors, then the original medical record was read through by the entire audit group. The audit group comprised senior obstetricians. However, when a more complete picture of normative care in other specialties was needed, the audit group consulted relevant specialists in cardiology, neurology, infectious diseases, and pathology. Special regard was taken to the year of the death since knowledge, prophylaxis, and standard treatment for some conditions, such as optimal blood pressure levels, thrombosis prophylaxis, eclampsia prophylaxis, and perimortem caesarean section have been modified over the course of the study period. The analysis and interpretation of sociocultural factors involved a medical anthropologist.

### Statistical analyses

Fisher’s exact test was used to compare suboptimal factors (major + minor) between Swedish-born and foreign-born women considering p-value <0.05 as statistically significant.

### Details of ethics approval

Ethics approval for this study was not needed according to Swedish laws on ethical review, since all women were deceased. The regional ethics committee in Uppsala, Sweden, confirmed that the study did not fall into the category of research requiring ethical clearance [2008/381, 2009-01-14]. All heads of clinical departments where a woman had been cared for were asked for consent to share a copy of the medical records.

## Results

### Cases of maternal death

No cases were excluded due to lack of consensus among the audit group. However, of the total maternal death cases, two Swedish-born cases were excluded as coincidental deaths. Of the remaining 73 cases, 13 women were foreign-born in LIC (Ethiopia, Eritrea, Somalia, Democratic Republic of Congo, Zimbabwe, Gambia, and Pakistan) and 12 in MIC (Poland, Former Yugoslavia, Turkey, Iran, Iraq, Morocco, Philippines, and Thailand). One foreign-born woman was an adoptee who had originated in a MIC but was brought up in Sweden from early childhood. The median age of all women was 32 years (range 21–45). For the foreign-born, the median age was 29 years (range 21–42), and for the Swedish-born 32.5 years (21–45). The case histories, including mode of pregnancy termination, are summarised in Table 1.

**Table 1:** Case histories, including mode of pregnancy termination, of maternal deaths in Sweden 1988–2010

	**Total (n = 73)**	**Foreign-born (n = 25)**	**Swedish-born (n = 48)**
Median age at death in years (range)	32.0 (21–45)	29.0 (21–42)	32.5 (21–45)
Died during first pregnancy or after first delivery	32	11	21
Early pregnancy death			
Miscarriage	8	4	4
Ectopic pregnancy	4	1	3
Mode of delivery			
Unassisted vaginal delivery	13	7	6
Vacuum extraction	6	2	4
Elective caesarean	2	1	1
Urgent/emergency caesarean	20	4	16
Peri-mortem caesarean^1^	12	4	8
Death during pregnancy			
<24 weeks	5	1	4
≥24 weeks	3	1	2
Place of death			
Outside hospital	4	1	3
Declared dead at hospital^2^	14	4	10
District hospital	6	2	4
County hospital	16	6	10
University hospital	33	12	21
BMI (kg/m^2)^			
<18.5	4	1	3
18.5–34.9	44	16	28
≥35	5	1	4
Information missing	20	7	13
Autopsy			
Clinical	38	8	30
Forensic	23	10	13
Relatives opposed	7	6	1
Not performed other reasons	4	0	4
Information missing	1	1	0

^1^During on-going resuscitation.

^2^Failed resuscitation during transport.

Overall, 52/73 cases were delivered by caesarean section, and 12 of those were performed during on-going resuscitation. In two attempted resuscitations the opportunity to perform perimortem caesarean was missed because it was not recommended at the time in Sweden (in 1996 and 2005) as an important part of resuscitation in pregnant women. Only 4 women died outside hospital whereas 14 had failed resuscitation for circulatory arrest during transportation and were dead upon arrival at the hospital. Information on BMI was missing in 20 cases. Overall, five women had BMI ≥ 35. If a death was sudden or could not be explained by the preceding clinical course of disease, or if the death could be suspected as associated with omissions or incorrect care treatment, a forensic autopsy should be performed. Among the 38 deaths that underwent a clinical autopsy, we identified conditions for which a forensic autopsy could have been performed, but was not. Autopsy was not performed at all in 11 cases. One case was missing information about autopsy. The majority of cases where relatives of the deceased woman opposed autopsy were foreign-born.

Table 2 shows causes of death by classification into direct (n = 37) and indirect (n = 35), and includes the number of cases having major and minor factors associated with suboptimal care. One Swedish case was assessed as a maternal death but it was not possible to classify direct or indirect status because the pathologist and clinicians involved in the case did not agree on the cause of death. The most common direct cause of death overall was amniotic fluid embolism (AFE); however no instances of major suboptimal care were evident in these cases. All remaining cases resulting from direct death had major contributing suboptimal care factors, including genital sepsis (3/5), venous thromboembolism (3/5), pre-eclampsia (3/4), and complications resulting from surgery (4/4). Both direct deaths resulting from haemorrhage were associated with major suboptimal factors of care. Among the overall indirect deaths, the majority resulted from cardiovascular conditions, which represent a heterogeneous group of diseases with a fairly even distribution of both major and minor suboptimal care factors. In all cases of non-genital sepsis, avoidable major suboptimal factors were identified, whereas courses of death from conditions of the CNS showed no associations to suboptimal care.

**Table 2:** Suboptimal factors associated with maternal death by cause of death in Sweden 1988–2010

**Causes of death**	**Total**			**Foreign-born**			**Swedish-born**		
	**n = 73**	**Cases with suboptimal factors (major + minor)**		**n = 25**	**Cases with suboptimal factors (major + minor)**		**n = 48**	**Cases with suboptimal factors (major + minor)**	
**Direct**	**37**	**28**	**(20 + 8)**	**16**	**14**	**(11 + 3)**	**21**	**14**	**(9 + 5)**
AFE^1^	7	5	(0 + 5)	2	2	(0 + 2)	5	3	(0 + 3)
Genital sepsis	5	4	(3 + 1)	2	1	(1 + 0)	3	3	(2 + 1)
VTE^2^	5	3	(3 + 0)	2	1	(1 + 0)	3	2	(2 + 0)
Pre-eclampsia^3^	4	4	(3 + 1)	2	2	(1 + 1)	2	2	(2 + 0)
Surgery^4^	4	4	(4 + 0)	3	3	(3 + 0)	1	1	(1 + 0)
Ectopic pregnancy	3	2	(1 + 1)	0	0		3	2	(1 + 1)
Haemorrhage^5^	2	2	(2 + 0)	2	2	(2 + 0)	0	0	
Other direct^6^	7	4	(4 + 0)	3	3	(3 + 0)	4	1	(1 + 0)
**Indirect**	**35**	**23**	**(16 + 7)**	**9**	**8**	**(4 + 4)**	**26**	**15**	**(12 + 3)**
Cardiovascular^7^	21	15	(8 + 7)	5	5	(1 + 4)	16	10	(7 + 3)
Non-genital sepsis	6	6	(6 + 0)	3	3	(3 + 0)	3	3	(3 + 0)
CNS^8^	6	0		1	0		5	0	
Other indirect^9^	2	2	(2 + 0)	0	0		2	2	(2 + 0)
**Unclear**^10^	**1**	**0**		**0**	**0**		**1**	**0**	

^1^Amniotic fluid embolism.

^2^Venous thromboembolism.

^3^Pre-eclampsia, eclampsia, HELLP syndrome.

^4^Complication of caesarean section (n = 2), complication of anaesthesia (n = 2).

^5^Cervix rupture (n = 1), postpartum haemorrhage (n = 1).

^6^Acute fatty liver of pregnancy (n = 1), peripartum cardiomyopathy (n = 2), anaemia (n = 1), unclear cause of death assessed as direct by the audit group (n = 3).

^7^Myocarditis (n = 3), myocardial infarction (n = 4), arrthymia/SADS (sudden arrhythmic death syndrome) (n = 3), aortic aneurysm (n = 8), rupture of splenic artery (n = 1), rheumatic valve disease (n = 1), congenital heart disease (n = 1).

^8^Central nervous system; intracranial haemorrhage (n = 5), epilepsy (n = 1).

^9^Pulmonary oedema (n = 1), ileus (n = 1).

^10^Assessed as a maternal death, however it is unclear if it was direct or indirect.

Four of the deaths were related to diseases originating in low-income settings. Two of these cases resulted from tuberculosis (TB), one case presented with severe anaemia, and one woman who had HIV died from rheumatic heart disease (discovered at autopsy). In both of the TB cases and the anaemia case, the women received major suboptimal care. The woman with rheumatic heart disease received minor suboptimal care.

### Factors of suboptimal care

In the majority of maternal death cases (51/73), suboptimal maternity care was associated with the mother’s death. In 36 of these, at least one major suboptimal factor was identified whereas in the remaining 15 cases, there were no major but at least one minor relevant factor was identified. Table 3 shows that there was a statistically significant difference in numbers of suboptimal factors, major and minor, between foreign-born and Swedish-born mothers. When calculating LIC and MIC separately, this statistically significant difference remained for women born in LIC, but not for the group of women born in MIC. Delay-causing factors were significantly different between the groups for care-seeking and receipt of quality medical care. However, there were no factors related to care accessibility among the Swedish-born deaths. All of the accessibility-related, delay-causing barriers were identified in the foreign-born group.

**Table 3:** Suboptimal factors associated with maternal death by phase of delay in Sweden 1988–2010. Fisher’s exact test (p < 0.05)

**Suboptimal factor**	**Total (N = 73)**		**Foreign-born (N = 25)**		**Swedish-born (N = 48)**		
	**Cases with suboptimal factors (major + minor)**^**1**^		**Cases with suboptimal factors (major + minor)**^**1**^		**Cases with suboptimal factors (major + minor)**^**1**^		**p value (Fisher’s exact test)**
**Total**	**51**	**(36 + 15)**	**22**	**(15 + 7)**	**29**	**(21 + 8)**	**0.01**
**Phase 1: Care-seeking**	**18**	**(6 + 12)**	**11**	**(1 + 10)**	**7**	**(5 + 2)**	**0.01**
Non-compliance	10	(3 + 7)	6	(1 + 5)	4	(2 + 2)	0.08 ns
Late-/non-booking	5	(0 + 5)	5	(0 + 5)	0		
Unhealthy lifestyle (substance abuse)	3	(3 + 0)	0		3	(3 + 0)	
**Phase 2: Accessibility of services**	**14**	**(3 + 11)**	**14**	**(3 + 11)**	**0**		
Limited language congruence	13	(3 + 10)	13	(3 + 10)	0		
Incomplete legal status^2^	2	(0 + 2)	2	(0 + 2)	0		
Delayed transport	1	(0 + 1)	1	(0 + 1)	0		
**Phase 3: Quality of medical care**	**50**	**(34 + 16)**	**21**	**(15 + 6)**	**29**	**(19 + 10)**	**0.03**
Inadequate care	49	(31 + 18)	21	(14 + 7)	28	(17 + 11)	0.02
Delay in consultation or referral^3^	24	(16 + 8)	10	(8 + 2)	14	(8 + 6)	0.2 ns
Appropriate care, but too late	16	(11 + 5)	5	(4 + 1)	11	(7 + 4)	0.5 ns
Miscommunication between providers	9	(5 + 4)	5	(3 + 2)	4	(2 + 2)	0.1 ns
Limited use/priority of resources^4^	5	(1 + 4)	2	(1 + 1)	3	(0 + 3)	0.6 ns

^1^The care on each level was assessed as major suboptimal if there was at least one major suboptimal factor, and minor if there were no major but at least one minor suboptimal factor. Note that the specified suboptimal factors within one level could add up to more than the total, because one woman could have several suboptimal factors within the same level, i.e. the numbers within each level could be more than by level.

^2^Recently arrived refugee (n = 1); lived in Sweden instead of Denmark due to Danish immigration law (n = 1).

^3^Referral from primary care to hospital, or between departments within a hospital.

^4^Lack of beds in intensive care unit (n = 4); delayed surgical procedure due to prioritisation of another patient (n = 1).

#### Phase 1: Care-seeking

No Phase 1 delay alone was assessed as a major or minor contributor to suboptimal care. However, among the foreign-born women, non-compliance was the most common Phase 1 delay to care-seeking. In the example below, Phase 1 factors were assessed as major for non-compliance (woman refused hospital care), Phase 2 factors were deemed minor, and the major contributing factor for Phase 3 was due to inadequate care (insufficient surveillance and delayed treatment).

##### Case A

A 21-year-old primigravida from the Middle East developed pre-eclampsia in gestational week 35 + 2. The patient declined hospital admittance because of communication problems, and was managed instead as an outpatient until the labour was induced. She delivered at hospital at week 36 + 2 with complications of atonic bleeding (2 L). Three days postpartum the woman requested discharge against the recommendations of the doctor. She was still hypertensive (140/100 mmHg), but was discharged and stayed with her baby at the neonatology ward. She was advised to come to the obstetric ward to record blood pressure, but no such check-ups were recorded. However, on Day 13, she presented at the obstetric ward as an emergency patient with heavy vaginal bleeding and had an immediate surgical evacuation of the uterus. The lowest Hb recorded, before transfusion, was 3.6 g/dL. One day later she had an irreversible cardiac arrest. Clinical autopsy showed heart infarction.

Most examples of Phase 1 delays leading up to the moment of death were minor contributing factors, such as failure to return for follow-up appointments. Other minor contributing factors included late-booking (after 20 weeks) for antenatal care (n = 3) and delayed care-seeking when an early pregnancy complication occurred (n = 2).

#### Phase 2: Accessibility of services

Limited language congruence created one or more Phase 2 barriers to accessing health care services among foreign-born women and their care providers. Where language incongruence was identified as the major barrier to accessibility (n = 3), the failure to communicate limited the woman’s ability to explain her health concerns to the care provider or from being forthcoming about additional care she had received elsewhere. The following example represents a death that was assessed as minor Phase 1 delay for non-compliance (withholding information), but major Phase 2 for limited language congruence and major Phase 3 for inadequate care (wrong diagnosis).

##### Case B

A 42-year old sub-Saharan African multipara, with two childbirths in Sweden but whose Swedish was recorded as poor, had an surgical evacuation of the uterus procedure planned at Hospital 1 for a miscarriage. The night before surgery she sought care at Hospital 2 for chest pains. No interpreter use was indicated. The doctor diagnosed her signs and symptoms as anxiety. During the morning of the surgical evacuation procedure at Hospital 1 she was assessed by an anaesthesiologist with help of a professional interpreter. As stated in the record, the patient said she was feeling well. However, there is no indication she mentioned about the emergency visit at Hospital 2 the night before. Immediately following the evacuation procedure, she had a cardiac arrest. Autopsy showed pulmonary embolism.

#### Phase 3: Quality of medical care

Inadequate care was the most common contributing factor to maternal death in both groups. However, among the foreign-born women, the influence of delay-causing barriers from Phases 1 and 2 could not be ignored. For example, out of 14 cases of major inadequate care, 10 cases also had Phase 2 barriers from, e.g. limited language congruence, and 8 of these also had Phase 1 barriers, including non-compliance or late-booking. The following example describes contributing factors as minor Phase 1 for late-booking, minor Phase 2 for limited language congruence, and major Phase 3 for inadequate care (delayed treatment) and delayed referral.

##### Case C

A sub-Saharan African woman who had given birth to one child in Sweden had lived in the country for more than four years, but was recorded as speaking poor Swedish. No interpreter use was documented. Antenatal care was booked late at gestation week 25. At that time the blood pressure was 110/60 mmHg. One week later, she presented at the same local clinic with nausea, abdominal and back pain, and a blood pressure of 155/85 mmHg. The next day she returned and tested positive for white and red blood cells, nitrite, and proteinuria (3+). Blood pressure was 130/70. She was prescribed treatment for a urinary tract infection and sent home. According to the investigation after her death, the woman had sought her general practitioner the day before her death, and again earlier on the same day, for headache. Sometime after her visit to the general practitioner but on the same day, she was admitted to the hospital ICU, had an eclamptic fit, and developed intracerebral haemorrhage. The patient was moved to the Neuro-ICU. Both treatment with magnesium and caesarean section were delayed for more than 12 hours because the obstetrician in charge did not attend the patient at the Neuro-ICU unit. Two weeks later the woman died.

Although inadequate care was more frequent among the foreign-born women, this was also the most commonly occurring barrier for the Swedish-born. The deaths that resulted from inadequate care included misdiagnosis, lack of reacting to typical symptoms, or insufficient surveillance. The influence of Phase 1 delays among the Swedish-born women included non-compliance and influence of unhealthy lifestyle.

##### Case D

A 23 year old Swedish woman relocated during an unplanned pregnancy from a rural Swedish community to live with her boyfriend in one of the major urban centres. The boyfriend had been imprisoned due to drug criminality and continued being criminal during the course of the pregnancy. No suspicion was ever raised that the woman was also using drugs. She had a cardiac arrest at 36 weeks, and the autopsy showed sepsis with staphylococcus aureus. Amphetamine was found in both hair and blood samples.

The Swedish women encountered no Phase 2 delays, including those that might occur in relation to receipt of optimal care in Phase 3. The remaining Phase 3 barriers to optimal care were not significantly different between the two groups. These included delayed consultation or referral, delayed receipt of appropriate care, and lack or prioritization of available resources. Examples of barriers between providers included transfer of information between specialists, for example between an obstetrician and anaesthesiologist, as well as failure to recognise needing assistance.

## Discussion

### Major findings

Almost all (69/73; 95%) women died at or on their way to a hospital. In two-thirds of cases suboptimal factors were identified. In half of these cases at least one major suboptimal factor contributed to the woman’s death irrespective of country of birth. Nevertheless, suboptimal care was a significantly more frequent contributing factor of maternal death for the foreign-born women than for the Swedish women. Among the foreign-born cases, many of these deaths were associated with communication-related barriers and delays to care-seeking. For example, failed ability to access clinical services was mainly due to language barriers and substandard interpretation services. The main delay to care-seeking was women’s non-compliance of care provider advice or treatment recommendations. At the medical facility, inadequate care occurred more often among the foreign-born, whereas delays in consultation/referral and miscommunication between providers showed no significant differences.

### Strength and limitations

A major strength of the study design is that the majority of cases were linked to three sources of register data: the Swedish cause of death register, the medical birth register, and the national patient register [5]. We further managed to obtain complete medical records for all but one foreign-born woman, who was excluded from the dataset. During the performance of the structured implicit review, our panel of clinical experts reached unanimous consensus per case while assessing the maternal death files, which strengthens validity [24]. We created a platform of normative knowledge that approximated the clinical context for when the maternal deaths occurred by conducting the audit with clinical experts who were employed as obstetricians during the time period of the dataset. The group of expert auditors might have included a wider base of obstetric specialists, e.g., midwives and obstetric anaesthesiologists. However, since the number of maternal death cases was small, it was preferred to consult with these specialists as the need arose. Similarly, experts from complementary specialities, i.e. cardiology and neurology, were consulted to fill gaps in knowledge about particular clinical events that occurred in the records. The introduction of a medical anthropologist was further necessary to broaden the holistic interpretation of the sociocultural aspects, and for expanding a theoretical base in maternal mortality research. These steps were necessary for applying the ‘migration three delays’ model, and for explaining how and why medical and social factors might relate to immigrant women’s experiences of maternity care in this HIC setting.

The main limitation is that our outcome interpretations are dependent upon a small sample size. However, these unique data are validated by having tracked the deaths nationally and by representing nearly completed record materials. The recruitment of cases represents an extended calendar period, which can be regarded as a limitation because both recording processes and clinical routines evolved over the study period. However, the long period of time was necessary in order to gain a utilisable sample size in this small population of Sweden. Additionally, the entrance of the foreign-born sample increased greatly during the later years of the study period, and constituted nearly half of the cases during the years 2006–2010. This potential limitation complicated the original matching procedure, but was overcome by matching two Swedish-born women who died before the index case. A weakness of the analysis and interpretation of sociocultural factors was the lack of interviews with professionals, as performed by the CMACE procedures or with relatives as performed during verbal autopsy, which could have minimised the risk for representation errors.

### Suboptimal quality of maternal care

Inadequate care included delayed or misdiagnosis. Several of these cases strongly suggest errors from premature satisfaction of the search, which is when the care provider becomes satisfied with an investigation once a single diagnosis is identified, even if it is not the root cause of the problem [28]. Other examples include inadequate and delayed treatment for hypertensive disorders and sepsis. This finding is consistent with substandard care reported from other European countries [8,29]. However, in the two cases that missed performing perimortem caesarean section, the audit group was unable to classify the providers’ limited knowledge about standards for resuscitation of pregnant women as suboptimal because the European guidelines were not yet published at the time the deaths occurred [30].

Priority of adequate consultation and referral is cited among the top-10 recommendations of the UK Confidential Enquiries into maternal deaths [8]. Enquiry into adverse maternal outcomes and patient safety has also shown that failures in teamwork can contribute to maternal death [7,8,31]. In our study, some level of provider-provider miscommunication contributed as a failure to optimal care. This includes information transfer between specialists but also between clinical departments. Furthermore, much focus has been on developing teamwork skills within a specific emergency situation, as in the delivery room [32]. Our results support that teamwork, both when taking care of a severely ill pregnant woman and when emergency situations arise in the labour room, should be multidisciplinary and include the timely entrance of complementary specialties, such as anaesthesia, cardiology, and surgical ICU [33]. Lack of intensive care beds was assessed as a factor contributing to four maternal deaths in this study, although we cannot say if it was a matter of de facto insufficient resources or insufficient communication between specialists, overview and triage of critically ill patients at a hospital level [34].

### Reliance on interpreter service

Interpreter use or the recording of interpreter usage was poor across the dataset despite that, over this time period, Swedish Law established the right to equitable health care for all inhabitants and that a patient has the right to a professional interpreter [35,36]. However, underuse of interpretation services was implied when the case file stated that the husband or other relative acted as translator during some component of the care leading up to the maternal death event. Standard guidelines for optimal medical interpretation services in a maternity care setting are limited [11]. Since the time period represented in this dataset, some European countries have implemented guidelines discouraging the use of personally known interpreters [8]. The implicit problem with lack of optimal interpretation services is that the language barriers between a woman and her care provider limit her ability to access and understand biomedical knowledge and similarly for the care provider to impart it [26]. Our findings are consistent with communication related problems for pregnant immigrant women in other western settings who experienced adverse birth outcomes [7,8,37-39]. In Sweden, one study also showed that lack of interpreter use contributed to perinatal deaths occurring between 1990 and 1996 [10].

### Negative responses to care-seeking

A number of cases related to care-seeking were influenced by such factors as late-booking and non-compliance. However, it is difficult to reconstruct a woman’s decision-making processes around her choices to seek care. The medical record can only document her registered contacts with a care centre, which become the perspective of the care provider, and from these we can presume whether or not a woman chose to comply. Sociocultural explanations for delays to care-seeking have been theorised as a ‘maternal migration effect’, where factors related to the pre-migration experience, have the potential to influence women’s obstetric choices after migrating to a new setting [27]. Other probable factors include mutual broken trust during the care encounter, which is also exhibited as women’s non-compliance/limited adherence to treatment advice and refusal of care as well as care provider frustration at not being able to impart quality care as a matter of course [26,40]. These negative approaches to care-seeking have the potential to delay the provision of optimal, preventive advice as well as timely referral. Mutual trust therefore requires that a woman’s care-seeking strategies are synonymous with the provision of trustworthy care [41].

### Causes of death

The most frequent causes of direct maternal death among the foreign-born women are comparable to those reported for maternal deaths across Europe [1,3,7,8,33,42]. Four women who died from diseases not usually seen in Sweden or Europe were from LIC, primarily sub-Saharan Africa. The association of limited professional insight into to those diseases illustrates the importance of improving knowledge about rare diseases complicating pregnancies, such as tuberculosis, rheumatic heart disease and severe anaemia, which have lately returned to European obstetrics [43-45]. Limited care provider knowledge about such pregnancy complications may also conflict with women’s limited insight into identifying risks to her pregnancy [27].

The importance of high-standard maternal autopsies to assure correct causes of death was emphasized in the latest UK maternal mortality report, and the recommendation is that maternal autopsies should be centralised [8]. We identified cases for which the quality of autopsy could be questioned. Some cases of sudden, unexpected death also fulfilled the criteria for forensic autopsy according to Swedish law, but then were not performed [46]. This suggests some clinicians have limited knowledge about laws regulating post-mortem examinations. Some of the relatives of the deceased women opposed autopsy. However, if the cause of death is unknown then the legal right to perform it remains intact even if the relatives oppose [47].

### Clinical implications

It is essential that maternal care providers are prepared to identify and meet the needs of all women, especially immigrant women, who may be at increased risk for maternal death or for experiencing other adverse pregnancy outcomes. This study emphasizes the obvious importance of using professional interpreters to avert adverse obstetric outcomes [8,10,11]. Regulations stating the right to professional interpretation for non-native speaking persons, such as those found in Sweden [35,36,47], should be incorporated into care management strategies across immigrant-receiving countries. Education of health care professionals should also not stereotype women’s care needs based upon presumed sociocultural barriers. Immigrant women want competent maternity care, just as all women, and the ‘culture-sensitive model’ needs to be founded on insight gleaned from research [11]. Gaining insight into the modified migration model used here could be useful to care professionals in other HIC settings for identifying context-dependent knowledge about any sociocultural factors relevant for immigrant women in their communities, and also for expanding sociocultural variables into maternal death reporting. Additionally, all patients receiving care in Sweden have the legal right to decline care [35]. However, limited guidelines exist for training professionals when it occurs [48]. Similarly, it is also important to identify management strategies for non-booking and non-compliance, and to appreciate that failure to comply is the problem of both women and care professionals. Western maternal care providers also need to increase their skills in recognition and interpretation of symptoms and appreciate that immigrant women may have other medical needs than those expected for host country women. Since suboptimal medical care contributes to many deaths that are simultaneously considered rare, it is important not to overlook ways to learn from adverse events and improve clinical management.

## Conclusions

Combining a maternal death audit procedure with a theoretical conceptual framework has helped to identify migration-related barriers to optimal care that are associated with maternal death in this high-income setting. The majority of maternal deaths were related to suboptimal care factors, but also to women’s care-seeking strategies and their limited ability to access quality maternity services. Immigrants from LIC and MIC are at increased risk of maternal death and this study advocates better insight into a migration perspective for clinicians who provide care to foreign-born women.

## Abbreviations

BMI: Body mass index; CMACE: Centre for Maternal and Child Enquiries; Hb: Haemoglobin; HIC: High-income country; HIV: Human immunodeficiency virus; ICD: International statistical classification of diseases and related health problems; ICU: Intensive care unit; LIC: Low-income country; MIC: Middle-income country; SFOG: Swedish Society of Obstetrics and Gynaecology; TB: Tuberculosis; UK: United Kingdom.

## Competing interests

The authors declare that they have no competing interests.

## Authors’ contribution

BE had the original idea. AE, PBF and BE prepared the protocol. AE and AML scrutinised the medical records. AE, BB, AML, UH and BE constituted the audit group. All co-authors participated in data analysis, and AE and PBF interpreted the data and wrote the manuscript in collaboration with the other co-authors. All authors have approved the final manuscript.

## Pre-publication history

The pre-publication history for this paper can be accessed here:

http://www.biomedcentral.com/1471-2393/14/141/prepub
